# Supplementary material for: Genome-wide mapping of genetic determinants influencing DNA methylation and gene expression in human hippocampus
Source: Nat Commun. 2017 Nov 15;8:1511. doi: 10.1038/s41467-017-01818-4 (PMC5688097; doi:10.1038/s41467-017-01818-4)
Supplement: Supplementary file 1 — Supplementary Information [file 41467_2017_1818_MOESM1_ESM.pdf]

SUPPLEMENTARY FIGURES

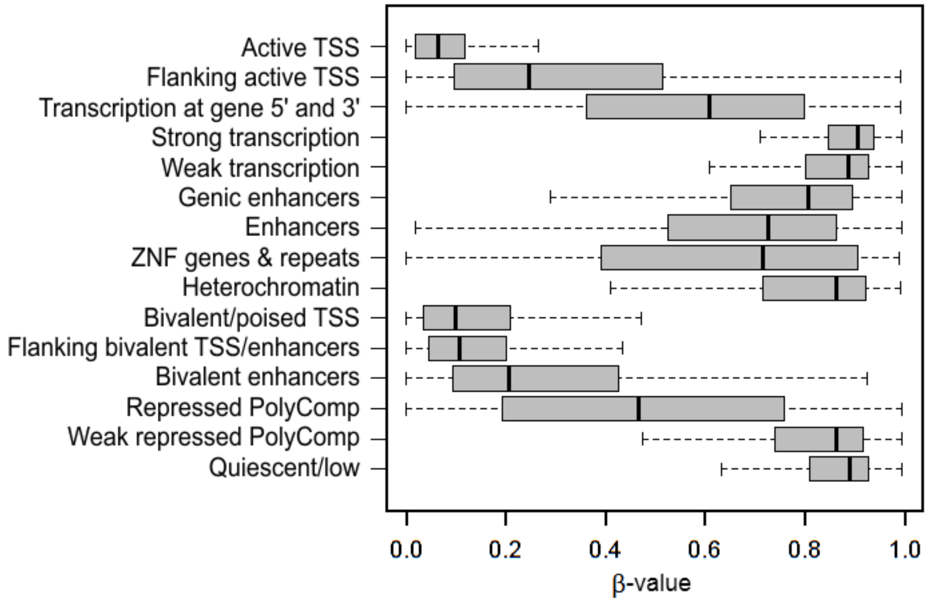

Supplementary Figure 1 | Degree of methylation projected on the Roadmap Hippocampus middle (E071) 15-chromatin-states[1].

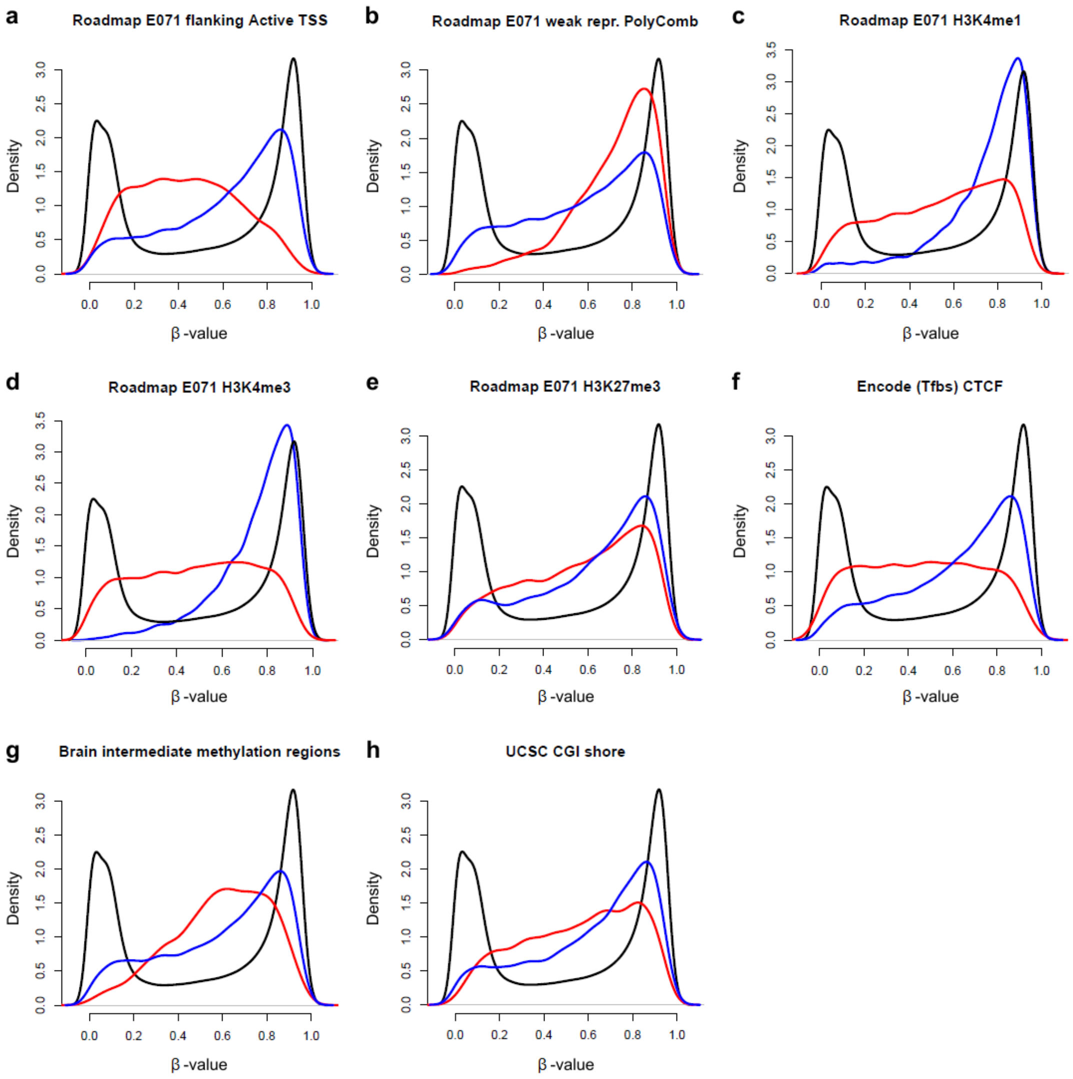

Supplementary Figure 2 | Distribution of methylation β-values for all 344,106 CpGs (black) compared to 14,118 *cis*-meQTL-CpGs in eight hippocampal functional epigenomic marks. The density of *cis*-meQTL-CpGs inside (red) and outside (blue) of functional epigenomic marks in the adult human hippocampus is plotted in respect to (a & b) the Roadmap[1] 15-core chromatin states: flanking active TSS and weak repressed PolyComb, (c–e) the histone marks H3K4me1, H3K4me3 and H3K27me3, (f) the ENCODE[2] hippocampal ChIP-seq peaks covering transcription factor binding sites of CTCF, (g) brain regions of intermediate DNA methylation[3], and (h) CpG-island shores.

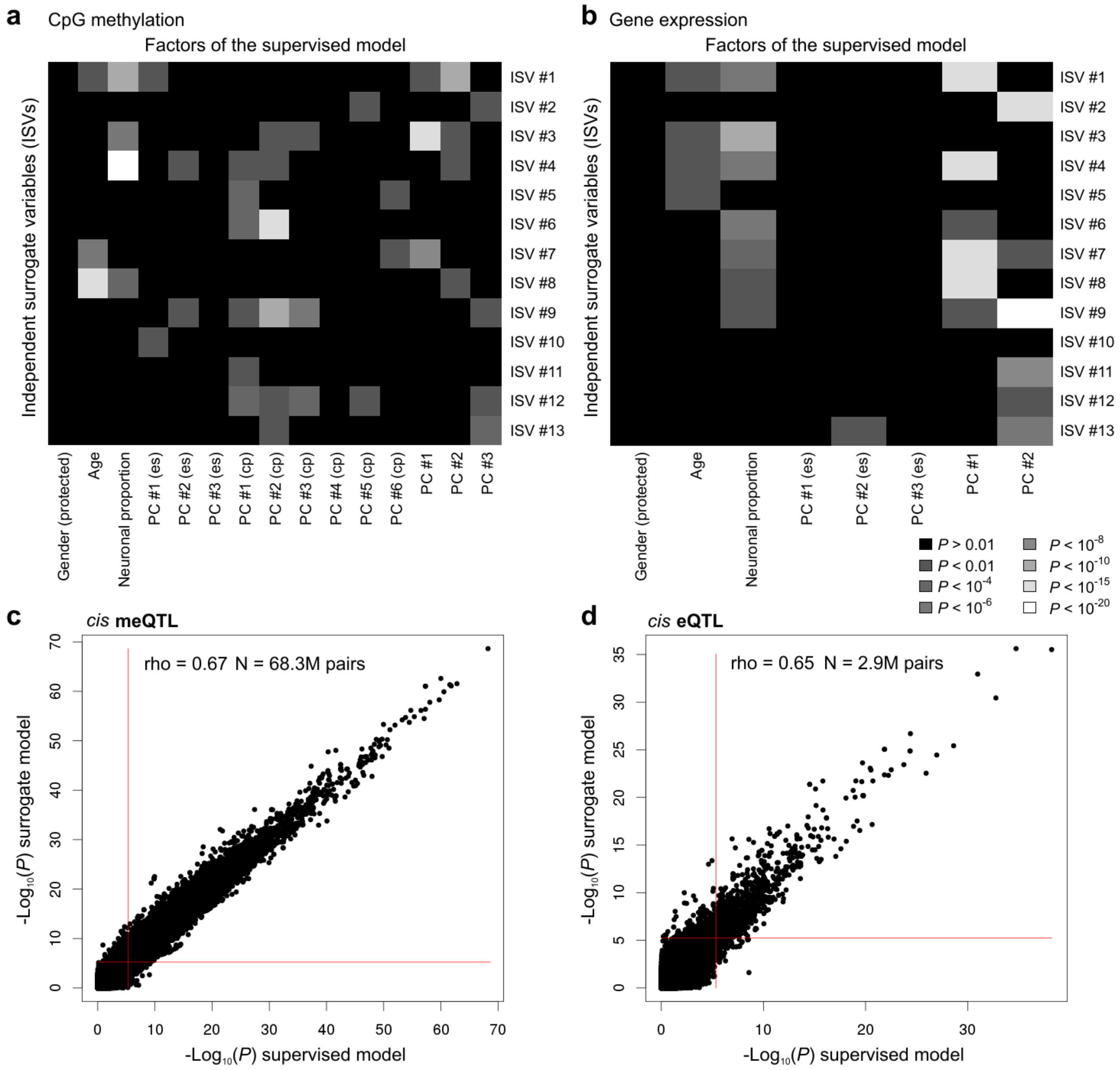

Supplementary Figure 3 | Correlation of the supervised covariates (a, b) with surrogate variables and comparison of significant meQTLs (c) and eQTLs (d) in a QTL analysis with ISV-adjustment[4]. (a & b) *P*-value matrix of associations between supervised covariates and inferred independent surrogate variables (ISVs). The variation covered by the determined IVSs is to a great extend represented in at least one of our defined covariates and principal components (PCs). (c & d) For *P*-values of both models, we observed a good Spearman correlation (rho between 6.5 and 6.7) of QTL *P*-values over all SNP/probe pairs (*cis*-frame of 0.5 Mb, n = 68,342,847 and n = 2,916,652 pairs in the meQTL and eQTL analysis). Moreover, 87.4% of the 66,970 significant (FDR of 1%) meQTL pairs and 88.7% of the 1,337 significant eQTL pairs reported in Supplementary Data 1 and 3 overlap with significant QTL pairs obtained in the ISV-adjusted QTL analyses. The supervised covariates do explain most of the variation that is captured by the ISV-adjustment. Legend: PC (es) = PCs components for population

stratification determined on the basis of genotypes using Eigenstrat; PC (cp) = PCs for batch effect correction determined on basis of array control-probes of the methylation dataset. PC = PCs determined on the basis of residuals of the cofactors age of surgery, gender, neuronal proportion, PC (es) and in the case of CpG methylation PC (cp).

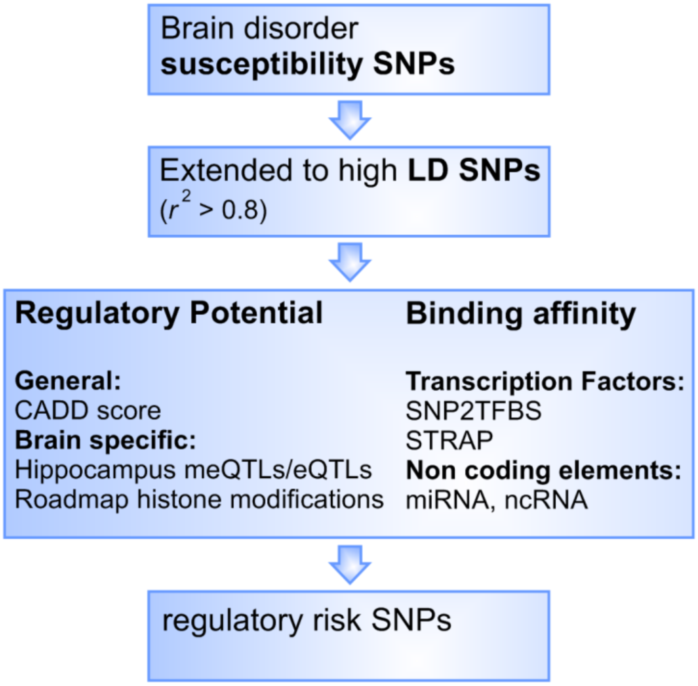

Supplementary Figure 4 | Functional epigenomic dissection of potentially causal regulatory SNPs. Flowchart indicating the step-wise filtering procedures to dissect and prioritize rSNPs conferring risk to brain disorders by epigenomic profiling of candidate SNPs derived from GWAS risk loci and candidate genes.

SUPPLEMENTARY TABLE

Supplementary Table 1 | Descriptive statistics on the phenotypes of the TLE patients in this study.

Number of mTLE patients	110
Gender (male vs. female)	52.7% vs. 47.3%
Age at seizure onset in years	13.1 ± 12.0
Age at epilepsy surgery in years	31.7 ± 16.9
Pathology (Ammon's horn sclerosis vs. lesion-associated)	67.3% vs. 32.7%
Drug therapy (Sodium-channel blockers monotherapy vs. levetiracetam combinations vs. non-levetiracetam combinations)	19.1% vs. 36.4% vs. 44.5%

Supplementary References

1. Kundaje A., *et al.* Integrative analysis of 111 reference human epigenomes. *Nature* **518**, 317-330 (2015).
2. The ENCODE Project Consortium. An integrated encyclopedia of DNA elements in the human genome. *Nature* **489**, 57-74 (2012).
3. Elliott G., *et al.* Intermediate DNA methylation is a conserved signature of genome regulation. *Nat. Commun.* **6**, 6363 (2015).
4. Teschendorff A. E., Zhuang J., Widschwendter M. Independent surrogate variable analysis to deconvolve confounding factors in large-scale microarray profiling studies. *Bioinformatics* **27**, 1496-1505 (2011).
