# Supplementary material for: Genome-wide mapping of genetic determinants influencing DNA methylation and gene expression in human hippocampus
Source: Nat Commun. 2017 Nov 15;8:1511. doi: 10.1038/s41467-017-01818-4 (PMC5688097; doi:10.1038/s41467-017-01818-4)
Supplement: Supplementary file 3 — Description of Additional Supplementary Files [file 41467_2017_1818_MOESM3_ESM.pdf]

Description of Additional Supplementary Files

File Name: Supplementary Data 1
Description: Hippocampal meQTLs (FDR of 1%)

File Name: Supplementary Data 2
Description: Enrichments / Depletions of cis-meQTL-CpGs in regulatory elements

File Name: Supplementary Data 3
Description: Hippocampal eQTLs (FDR of 1%)

File Name: Supplementary Data 4
Description: Hippocampal eQTMs (FDR of 1%)

File Name: Supplementary Data 5
Description: Hippocampal meQTL SNP consequences

File Name: Supplementary Data 6
Description: Hippocampal eQTL SNP consequences
